# Regulation of the decision threshold by the locus coeruleus

**DOI:** 10.1038/s41386-026-02399-x

**Published:** 2026-04-13

**Authors:** Hongjie Xia, Maxime Maheu, Gary A. Kane, Benjamin B. Scott

**Affiliations:** 1 Department of Biology, Boston University, Boston, MA USA; 2 Département d’Études Cognitives, École Normale Supérieure, Université PSL, Paris, France; 3 Department of Psychological and Brain Sciences, Boston University, Boston, MA USA; 4 Center for Systems Neuroscience, Boston University, Boston, MA USA

**Keywords:** Decision, Perception

## Abstract

A fundamental challenge for decision-making under uncertainty lies in balancing speed and accuracy. Humans and animals solve this problem by adjusting decision thresholds—the criterion that determines how much information is required before committing to a choice. While brain regions associated with this process have been identified, the neural circuits that *directly* alter decision thresholds remain unknown. Here, we investigate the role of the locus coeruleus (LC) norepinephrine (NE) system in controlling this balance. Through cell-type-specific chemogenetic manipulations, we discovered that LC-NE activation increased decision thresholds. This effect is replicated by administration of the α2-adrenergic receptor (α2-AR) agonist clonidine. Notably, α2-AR activation altered decision threshold specifically, without reproducing other LC-NE activation effects such as promoting task engagement. Together, these results suggest that LC-NE regulates decision thresholds, possibly via downstream α2-ARs.

## Introduction

A core feature of adaptive behavior is the ability to make decisions under uncertainty. In this situation, humans and other animals face a fundamental challenge: deciding when to terminate deliberation and commit to a choice. Prioritizing speed can yield error-prone decisions, whereas emphasizing accuracy delays outcomes. This balance, known as the speed-accuracy tradeoff, is a cornerstone of human and animal behavior in uncertain and time-sensitive environments [1, 2].

Conceptually, a balance between speed and accuracy can arise from adjustments in decision threshold, defined as the amount of evidence required before making a choice, with higher thresholds yielding slower and more accurate choices [3]. Past research into decision thresholds has largely focused on the neural correlates of decision commitment in cortical and subcortical regions [4–8]. However, the circuits that alter the threshold remain unresolved. One candidate is the brainstem locus coeruleus (LC) norepinephrine (NE) system, a circuit known to play an important role in behavioral flexibility [9–11]. Here, we tested the idea that the LC-NE is involved in controlling the threshold for decisions, effectively shifting the balance of speed and accuracy during decision-making.

The locus coeruleus is a small, bilateral nucleus located in the dorsal pons of the hindbrain and serves as the brain’s principal source of norepinephrine. Despite its small size, the LC sends widespread axonal projections to cortical and subcortical regions [12], positioning it as a key modulator of brain-wide activity supporting adaptive decision-making [13–16]. To date, the involvement of the LC-NE system in regulating decision thresholds has been inferred from either pupillometry or pharmacological manipulations in humans [17, 18]. Yet pupillometry is not LC-specific [19–21], and pharmacological agents influence catecholamine signaling broadly [22]. Consequently, direct evidence for the role of LC-NE in regulating the decision threshold is still lacking.

In this study, we directly investigated how the LC-NE system shapes the balance between speed and accuracy during decision-making under uncertainty. We used chemogenetic manipulations of the LC-NE system in rats performing a behavioral task designed to offer a tradeoff between speed and accuracy. LC activation resulted in slower and more accurate decisions, consistent with elevated decision thresholds. These effects were replicated by administration of a selective α2-adrenergic receptor (α2-ARs) agonist, clonidine. Together, these findings suggest that LC-NE activation promotes more deliberative decision-making strategies, possibly via α2-AR signaling.

## Methods

### Animals

All experiments and procedures were performed in accordance with protocols approved by the *Boston University Animal Care and Use Committee*. Adult male and female Long-Evans rats (*Charles River Laboratory*; criver.com) aged 3–6 months were used. Rats were food-restricted and maintained above 80% of baseline weight throughout the training period. Animals were weighed weekly, and their mean body weight across the study was 109.2% of baseline (SD = 8.06%). Rats were housed in 12 h light-dark cycle. All behavioral training was conducted during the light period.

### Behavioral task

**Fig. 1:**
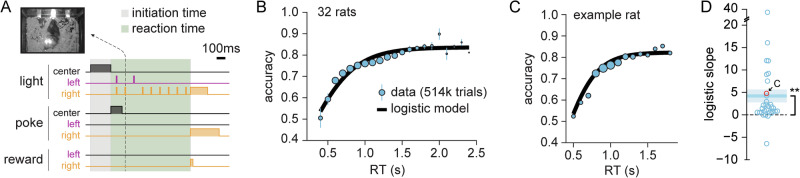
Rats trade between speed and accuracy in a free-response perceptual task. **A** Schematic of the timing of task events in an example trial, including lights, pokes, and reward delivery. Each trial begins with an initiation period during which the center light is illuminated. A center poke initiates presentation of a series of flashes from the left and right LEDs. The flash sequence ends when the rat makes a choice by poking into a side port (here, right). On correct trials, the corresponding side light illuminates, and a reward is delivered. Initiation time (shaded gray) is the interval from the center light onset until the center port is poked. Reaction time (shaded green) is the interval from center port nose poke until the animal commits to a choice by poking a side port (see “Methods”). **B** Chronometric function reporting accuracy as a function of reaction times (RT; 100 ms bins; blue dots) for rats (*n* = 32; mean ± SEM). Dot size is indexed on the proportion of trials in each RT bin. The black line shows the prediction of a logistic regression model fitted to rat behavior. **C** Accuracy across trials with different reaction times (RT) from an example rat. Data is shown as blue dots, regression fit as black line. **D** Slope parameter from the logistic regression model shown in (**B**, **C**). A positive parameter value indicates that accuracy increases with RT. Each dot corresponds to one rat. The rat shown in (**C**) is indicated with a red circled dot. One-sample t-test, **p < 0.01.

Rats performed the task in a customized operant chamber for 2 h daily, from Monday to Friday. Each chamber contains three nose ports equipped with a white LED. This task has been previously described [23, 24]. To briefly summarize here, rats begin each trial by poking their nose in the center port. This triggers a first bilateral flash followed by a 10 Hz sequence of 10 ms unilateral flashes, occurring from either the left or the right port. The occurrence of flashes followed a Bernoulli process with a 75% probability of a flash occurring on the correct side and a 25% probability on the incorrect side. The correct and incorrect side is randomly selected on each trial. Rats report their choice by poking to either side port at any time during the trial. Following correct trials, rats receive 25 μl of a 10% sucrose solution and are imposed a 5 s inter-trial interval. Following incorrect trials, rats receive a 3 s timeout punishment, leading to a total of 8 s inter-trial interval. If rats fail to respond within 8 s following trial initiation, the trial is considered an “omission”. In practice, omitted trials were rare (1.09%). A total of 32 rats (16 females) performed the task without chemogenetic or pharmacological manipulations (Figs. 1 and S1), yielding a dataset of 514,978 trials.

### Behavioral training

In the first stage of training, rats are rewarded by poking the illuminated side port. In stage 2, rats are rewarded by first poking into the center port, then to the illuminated side port. In stage 3, all flashes appear on the correct side, and rats received a reward after poking on that side. In stage 4, the probability of flashes occurring on correct vs. incorrect is set to 90%–10%. In stage 5, this probability is set to 80–20%. Stage 6 is the final stage and corresponds to a probability of 75–25%. All trials in this study were collected in stage 6.

### Surgery

**Fig. 2:**
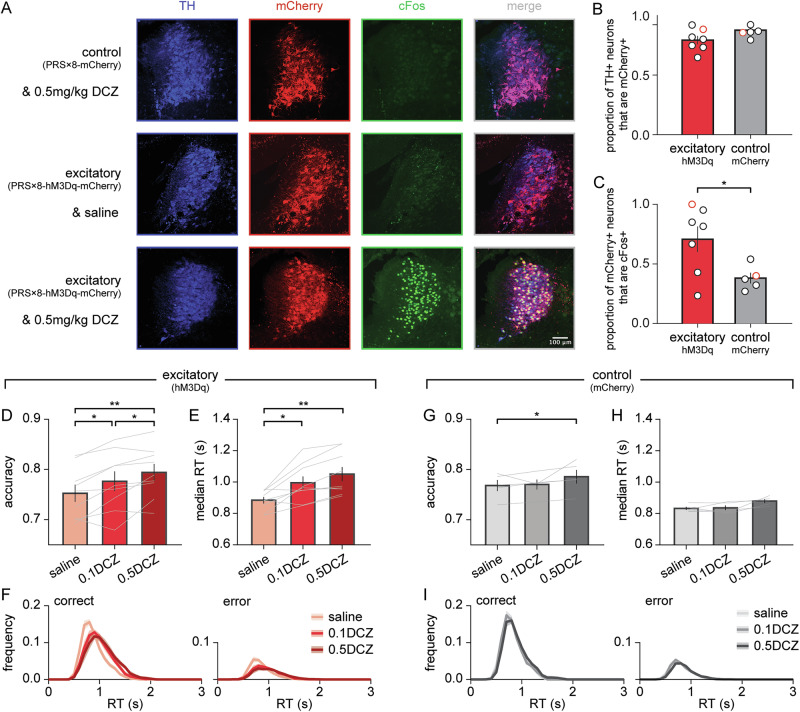
Chemogenetic activation of LC-NE increases accuracy and reaction time. **A** Immunofluorescence images of the LC. Top row: control group (PRS×8-mCherry) following injection of 0.5 mg/kg DCZ. Middle row: excitatory DREADD group (PRS×8-hM3Dq-mCherry) following saline injection. Bottom row: excitatory DREADD group following 0.5 mg/kg DCZ injection. TH tyrosine hydroxylase. **B** Proportion of TH^+^ neurons expressing the transgene (mCherry). Example rats shown in (**A**) are highlighted in red circles. **C** Proportion of TH^+^ and mCherry^+^ neurons expressing cFos after DCZ injection. Unpaired *t*-test, *t*_(10)_ = 1.155, *p* = 0.035. Example rats shown in (**A**) are highlighted in red circles. Behavioral effects in the excitatory DREADDs group (PRS×8-hM3Dq-mCherry, *n* = 9). **D** Mean accuracy ( ± SEM). Each line represents one animal. Paired *t*-tests with Bonferroni correction, saline vs. 0.1 DCZ: *t*_(8)_ = 3.088, *p* = 0.045; saline vs. 0.5 DCZ: *t*_(8)_ = 4.461, *p* = 0.006. *p < 0.05,**p < *0.01*. **E** Median reaction time (mean ± SEM) across animals. Each line represents one animal. Paired *t*-test with Bonferroni correction were performed for saline vs. 0.1DCZ: *t*_(8)_ = 3.431, *p* = 0.027; saline vs. 0.5DCZ: *t*_(8)_ = 4.316, *p* = 0.008; and 0.1DCZ vs. 0.5DCZ: *t*_(8)_ = 3.922, *p* = 0.013. *p < 0.05, **p < *0.01* **F** RT distributions in correct (left) and error (right) trials. Mean ± SEM. Bin width = 0.1 s. Control group, PRS×8-mCherry (*n* = 5). **G** Mean accuracy ( ± SEM). Each line represents one animal. One-way ANOVA, *F*_(2,8)_ = 4.939, *p* = 0.040. Paired *t*-test with Bonferroni correction, saline vs. 0.5DCZ, *t*_(4)_ = 5.774, *p* = 0.013. *p < 0.05 for pairwise comparison. **H** Median reaction time (mean ± SEM). Each line represents one animal. One-way ANOVA, *F*_(2,8)_ = 5.607, *p* = 0.030. **I** RT distributions in correct (left) and error (right) trials. Mean ± SEM. Bin width = 0.1 s.

A total of 14 rats (6 females) were included in the chemogenetic experiment. Once rats reached the final stage of training, they received a bilateral virus injection in the LCs. CAV-PRSx8-hM3Dq-mCherry was obtained from the *Plateforme de Vectorologie de Montpellier* (plateau-igmm.pvm.cnrs.fr). As CAV-PRSx8-mCherry was not available at the time of the experiments, adeno-associated virus AAV9-PRSx8-mCherry was obtained from the *UPenn Viral Core*. The surgical procedure started by anesthetizing the rat with isoflurane (5% induction, 1-2% maintenance). For analgesia, buprenorphine (0.02 mg/kg, intraperitoneal) was administered. Rats were aligned in the stereotaxic frame such that lambda was 2 mm above bregma (corresponding to 15-20° head angle). Bilateral burr holes were drilled at the following coordinates: AP (from lambda): −3.9 mm, ML: ±1.35, and viruses were injected at a depth of 6-6.5 mm from the surface of the brain. Rats received AAV9-PRS×8-mCherry (control), CAV-PRS×8-hM3Dq-mCherry (excitatory group, 5.7 × 10^12^ pp/mL before dilution, 1:6 diluted with sterile PBS). We quantified viral efficacy as the percentage of tyrosine hydroxylase (TH)–positive LC neurons that were labeled by the viruses. Using this metric, CAV-PRSx8-hM3Dq-mCherry labeled 75.74% of TH-positive neurons, while AAV9-PRSx8-mCherry labeled 87.58% of TH-positive neurons (Fig. 2A, B). Viral specificity was quantified as the proportion of virally labeled neurons that were TH-positive AAV9-PRSx8-mCherry showed a specificity of 90.72%, whereas CAV-PRSx8-hM3Dq-mCherry showed a specificity of 81.52% (Fig. S2). These values are higher than those previously reported in mice [25], which may reflect differences in species, viral serotype, or injection parameters. A total volume of 1 μl was injected into each LC at a rate of 100nL/min using a Hamilton syringe. Rats were given 7 days to recover from surgery (before behavioral sessions resumed), during which they were given *ad libitum* access to food.

### DCZ administration

DREADDs were activated by deschloroclozapine dihydrochloride (DCZ), which was chosen for its high selectivity for DREADDs [26]. We obtained DCZ through the *NIMH Chemical Synthesis and Drug Supply Program*. DCZ was diluted in sterile saline with a stock concentration of 0.5 mg/mL. The stock solutions were kept at −20 °C. DCZ stock solution was further diluted to 0.1 mg/mL in order to obtain the desired 0.1 mg/kg dosage. Working concentrations were kept at room temperature for up to 4 days. To habituate rats to injections, we injected rats with saline during the first week of training. Then, rats were dosed on alternating days in an interleaved sequence (saline → 0.1 mg/kg DCZ → 0.5 mg/kg DCZ → saline → 0.1 mg/kg DCZ…), each administered i.p. 10 min before the behavioral session. A total of at least five sessions for each concentration were collected from each rat.

### Histology

Rats were euthanized with sodium pentobarbital euthanasia solution at a dosage of, or greater than, 200 mg/kg. Rats were perfused with 10% neutral buffered formalin one and a half hours after the start of the final training session. Brains were extracted and postfixed with 10% formalin for at least 72 h before sectioning. Brains were sliced at 40 µm thickness using a vibratome. 1 in 3-series sections were incubated overnight with primary antibodies diluted in 2% non-fat milk and 0.1% Triton X-100 in 1xPBS. To confirm the increased cell activity following DCZ injections in hM3Dq-expressing animals, brain slices were co-immunostained with a rabbit anti-cFos antibody (*Abcam*, ab190289, 1:500) and a mouse anti-TH antibody (*Immunostar*, 22941, 1:1000). On the next day, after three washes of 1xPBS, slices were incubated with secondary antibodies at room temperature for 2 h with agitation. Goat anti-mouse 647 (*Abcam*, ab150115), goat anti-rabbit 488 (Invitrogen, A-11008), and goat anti-mouse 568 (*Invitrogen*, A-11004) secondary antibodies were used during this process. Finally, slices were washed three times with 1xPBS and mounted onto microscope slides.

### Image acquisition and cell counting

All confocal images were taken using a *Nikon C2 Si* confocal microscope. Z-stacks were acquired, and channel-specific maximum projection images were obtained using *ImageJ*. Images were then converted into 8-bit gray scale and imported into *CellPose 2.0* for segmentations [27] (github.com/MouseLand/cellpose). For each channel, 50% of the images were manually labeled, and a new model was trained using these manually defined cell masks. This model was then applied to the other half of the images. Missing or false positive cell masks were manually curated. Cell masks were opened with *ImageJ*’s ROI manager for counting cells. In order to count overlapped cell masks in any two channels (TH^+^ and cFos^+^ on one hand, and TH^+^ and mCherry^+^ on the other hand), cell masks from each channel were first “Combine(OR)” into a single cell mask, and overlapped cell masks were created between two channels using the “AND” function. The total number of cell masks and corresponding fluorescence values was exported for further analyses using custom-made scripts, and cell masks smaller than five pixels were eliminated.

### Clonidine administration

A total of 6 female rats were included in this experiment. Clonidine hydrochloride was first dissolved in sterile saline at a 1 mg/mL concentration, then aliquoted and stored at −20 °C (*Tocris*, cat no. 0690). Clonidine was further diluted into a final injection volume of 1 mL/kg weekly before usage and stored at room temperature. Rats received intraperitoneal injections of either saline or clonidine (5, 10, 20, or 50 μg/kg) on interleaved days. Injections occurred 10 min prior to the start of the behavioral session.

### Trial exclusion

Prior to all behavioral analyses, we excluded trials with reaction times (RT) shorter than 0.3 s, indicative of false nose port entries (due to photogate instability). In addition, we excluded the 10% of the trials associated with the longest RTs on an individual basis, with the idea that behavior in these trials did not reflect stimulus-driven decisions, but often corresponded to unintended initiation.

### Logistic regression model

A logistic regression model explaining accuracy as a function of RT is defined as:

$$P\left({y}_{i}={\mbox{correct}}|{\mbox{RT}_{i}}\right)=w\cdot \frac{1}{1+\exp \left(-a\cdot {{\mbox{RT}}}_{i}+b\right)},$$

where ${y}_{i}$ is the outcome at trial $i$. Free parameters $(w,{a},{b})$ are fitted using the *sklearn* package in *Python* and the slope parameter ($a$) was tested against zero to test for the existence of a speed-accuracy tradeoff in rats

### Drift diffusion model

We accounted for rats’ behavior using a vanilla drift diffusion model (DDM), which casts decision-making as a stochastic process in which a decision particle drifts over time until a decision threshold is reached. The DDM has 4 free parameters: drift rate ($v$), decision boundary (*a*), non-decision time (${t}_{0}$) and starting point ($z$) [28, 29]. The DDM considers that the stochastic drift follows a Wiener diffusion process:

$$\mbox{d}X\left(t\right)=v\cdot {\mbox{d}}t+\sigma \cdot {\mbox{d}}W\left(t\right),$$

where $X\left(t\right)$ is the decision variable at time $t$ and $\sigma$ is the noise scaling factor. In this model, a decision is made when $X\left(t\right)$ reaches one of two boundaries. The lower boundary is set to 0 and the upper boundary is equal to $a$. The starting point $z$ is defined as a proportion of the boundary separation, such that an unbiased process corresponds to $z=0.5$. Reaction time (${{{\rm{RT}}}}$) is composed of decision time (${{{\rm{DT}}}}$) and non-decision time (${t}_{0}$), with the latter capturing processes peripheral to the decision process, such as sensory encoding and motor execution:

$${{{\rm{RT}}}}={{{\rm{DT}}}}+{t}_{0}.$$

Free parameters are fitted using a custom *R* package (github.com/gkane26/rddm). Parameters are estimated separately for each rat and each condition using the *qmpe* package, which compares observed and simulated RT distributions using a quantile maximum estimation method [30]. To confirm the recoverability of the DDM, we generated 50 independent sets of parameters by drawing each parameter from a normal distribution, with mean and standard deviation corresponding to the group average from the saline condition. With 2000 trials per simulation, similar to those contributed by each rat per condition, the DDM’s free parameters were recoverable (Fig. S4).

### Dual-state model

In the dual-stage model, choice outcome is modeled as a mixture of an engaged, logistic process and a stimulus-independent, lapse process. In different versions of the model, choice outcome probability in the engaged state is a function of either stimulus strength (balance between number of left vs. right flashes; see Fig. S1) or RT. In all cases, the probability of a correct choice on trial $i$ is given by:

$$P\left({y}_{i}={\mbox{correct}}|{x}_{i}\right)=\left(1-\lambda \right)\cdot \frac{1}{1+\exp \left(-a\cdot {x}_{i}+b\right)}+0.5\cdot \lambda ,$$

where ${x}_{i}$ is the predictive variable (stimulus strength or RT), $a$ is the slope, and $b$ is the intercept of the logistic function. Lapse trials occur with probability $\lambda$ producing random choices independent of ${x}_{i}$.

### Statistical analysis

ANOVAs and paired *t*-test were performed using the *pingouin* package in *Python*. To control for family-wise type 1 error, the Bonferroni correction was used to adjust *p*-values. Normality of paired differences was assessed using the Shapiro-Wilk test. For initiation time, most conditions significantly deviated from normality (*p* < 0.05); therefore, we used non-parametric Wilcoxon signed-rank significance tests.

## Results

### Rats trade between speed and accuracy in a free-response perceptual task

We trained rats in a perceptual task designed to elicit a tradeoff between speed and accuracy [24]. In this task, reward location is indicated by a sequence of light flashes distributed between the left and right nose ports, with the rewarded side having a higher probability of flashes (75% congruent; Fig. 1A). Animals are allowed to respond at any time during the presentation of the flash sequence by poking into either the left or right nose port. Because each flash provides uncertain evidence about the rewarded side, rats can increase their accuracy by sampling more flashes.

We used high-throughput operant techniques to collect large-scale data (>514,000 trials total) from rats (*n* = 32) performing this task. Rats performed this task at high accuracy (mean=75.8%, range = 68.4–82.8%, s.d. = 0.7%) and moderate RTs (median 0.95 s, range = 0.72–1.14 s, s.d. = 0.14 s). Importantly, most rats showed a positive relationship between accuracy and RT, such that waiting longer improved performance (Figs. 1B–D and S1). These data indicate that rats exhibit a speed–accuracy trade-off in this task, in which longer RTs allow the integration of evidence from multiple flashes.

### Chemogenetic activation of LC-NE shifts the speed-accuracy tradeoff

We tested whether the LC-NE system regulates rats’ speed-accuracy tradeoff in this task using a chemogenetic approach. We injected viruses carrying excitatory DREADDs (hM3Dq) under a synthetic promoter selective to NE neurons (PRS×8 [31]) into the LC bilaterally (see *Methods*).

We first validated the approach by confirming selective transduction of TH^+^ neurons in the LC increased expression of the immediate-early gene cFos, a proxy for neural activation (Fig. 2A, B). Injection of deschloroclozapine (DCZ) [26] caused a significant increase in cFos levels in rats with excitatory DREADDs in comparison to a separate group of rats expressing a control transgene (PRS×8-mCherry; Fig. 2C). These data are consistent with previous studies reporting increased LC spiking activity following PRSx8-hM3Dq stimulation in rats [32].

Behaviorally, chemogenetic activation of LC-NE neurons simultaneously slowed RT and improved accuracy in the task in comparison to control saline injections (Figs. 2D–F and S3). These effects were observed at 0.1 mg/kg DCZ and further increased after 0.5 mg/kg DCZ administration (Figs. 2D–F and S3).

By contrast, no changes in accuracy or RT were observed in the group of rats expressing the control transgene (PRS×8-mCherry) after administration of 0.1 mg/kg DCZ (Fig. 2G-I).

However, we noted that at 0.5 mg/kg DCZ, there was a modest change in RT and accuracy (Fig. 2G, H), suggesting that high doses of DCZ may produce a marginal behavioral effect independent of hM3Dq activation. Nevertheless, these effects were smaller than the effects of stimulation with hM3Dq (Fig. S2). Together, these data indicate that activation of LC-NE neurons promotes slower and more accurate decisions, reflecting a shift in the tradeoff between speed and accuracy.

### Activation of LC-NE increases boundary separation

**Fig. 3:**
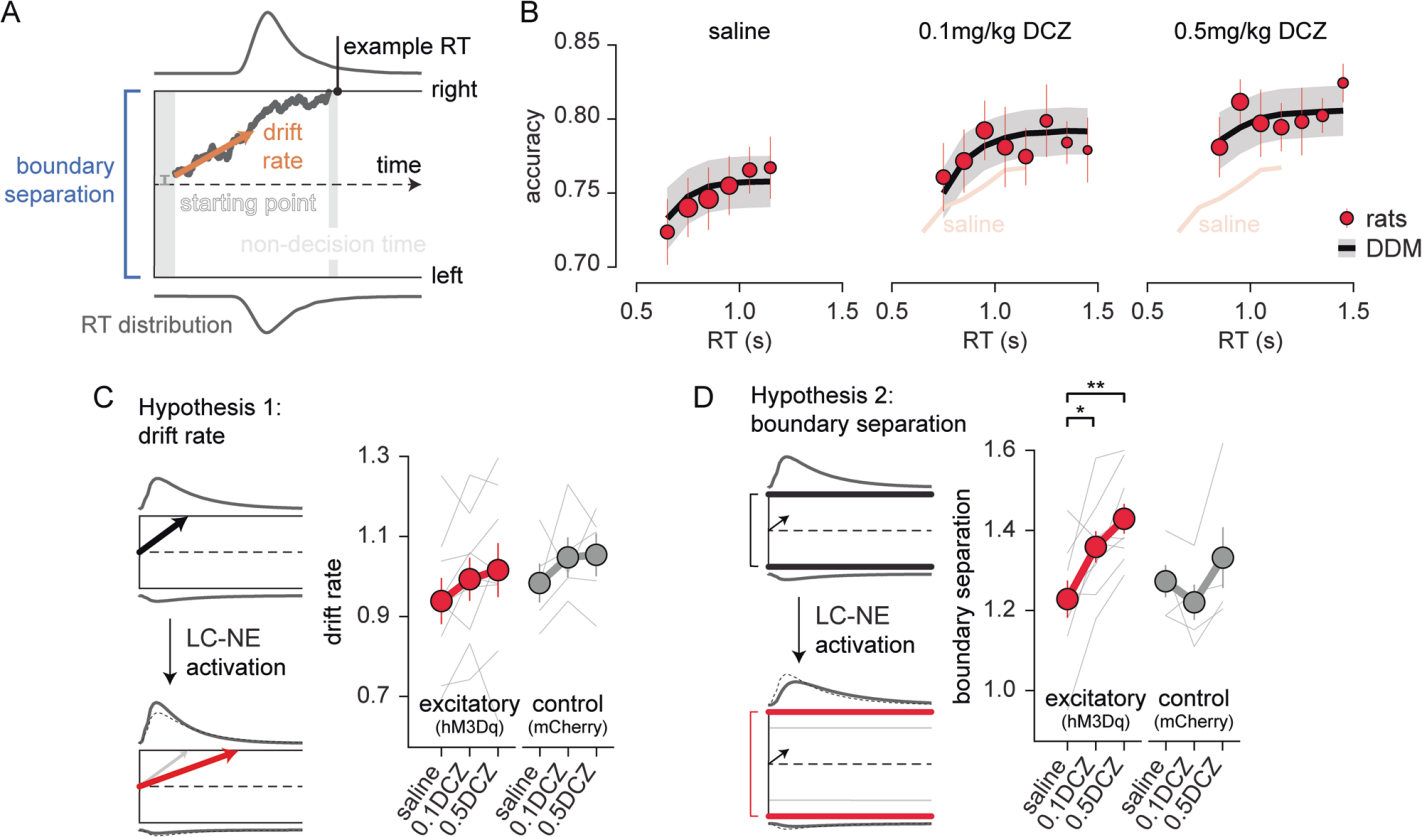
Chemogenetic activation of LC-NE increases boundary separation. **A** Schematic of the drift diffusion model (DDM) and its four free parameters: drift rate, boundary separation, starting point bias and non-decision time. **B** DDM fit of behavioral data from the excitatory group. Red dots indicate rat accuracy (mean ± SEM) within each RT bin. Black lines show fitted DDM predictions (mean ± SEM). **C** Illustration of potential changes in drift rate within the DDM (left). Mean fitted drift rate ( ± SEM) for the excitatory and control groups (right). **D** Illustration of potential changes in boundary separation within the DDM (left). Mean fitted boundary separation ( ± SEM) for the excitatory and control groups (right). Paired *t*-tests with Bonferroni correction, saline vs. 0.1 mg/kg DCZ: *t*_(8)_ = 3.587, *p* = 0.021, and saline vs. 0.5 mg/kg DCZ: *t*_(8)_ = 5.598, *p* = 0.002. *p < 0.05, **p < 0.01.

To directly determine if LC-NE activation alters decision threshold, we accounted for rat behavior using a drift diffusion model (DDM; Fig. 3A). The DDM conceptualizes decision-making as a stochastic process in which a decision variable drifts towards one of two decision boundaries under the influence of the stimulus [28, 29, 33]. The DDM provides a unified account of both accuracy and RT and, as such, is a widely adopted framework for describing shifts in the speed-accuracy tradeoff [34].

Free parameters in the DDM include the drift rate, which captures the rate at which the sensory evidence influences the decision variable; and boundary separation, which sets the distance between the two decision thresholds [35] (for left and right decisions; see “Methods”) (Fig. S4).

We fitted the DDM separately to data from different experimental conditions and inspected possible changes in model parameters. The DDM captured the speed-accuracy tradeoff in this task well (Fig. 3B). DCZ injections in excitatory DREADDs-expressing rats produced a significant increase in boundary separation in comparison to saline injections; an effect that was DCZ dose-dependent (Fig. 3D, E). In contrast, the drift rate parameter remained mostly unaffected by DCZ injections (Fig. 3C). DCZ injections in the group of control (mCherry) rats did not result in significant changes in any of the DDM parameters (Figs. 3C, D and S5).

Next, we considered the alternative hypothesis that the effect of LC-NE activation may reflect a shift in attention, not a change in decision threshold [36]. To test this hypothesis, we fitted rats’ decisions with a dual-state model, which considers that choices are either informed by the flash sequence or made randomly (see “Methods”). Experimental manipulations did not alter the proportion of stimulus-independent decisions, which remained around 5% (Fig. S6).

Together, these data are consistent with a model in which LC-NE neurons regulate decision thresholds, rather than processes peripheral to the decision itself, such as the sensitivity to sensory evidence (as captured by DDM’s drift rate) or attentional states (as captured by a dual-state model).

### Activation of α2-ARs increases accuracy, RT and boundary separation

Our previous experiments demonstrated that LC-NE activation causes a shift in rats’ speed-accuracy tradeoff consistent with an increase in decision threshold. However, an open question concerns the mechanisms mediating this shift. LC-NE neurons release NE as well as other factors, which are known to bind to different receptor subtypes [37]. Amongst these subtypes, α2 adrenergic receptors (α2-ARs) have the highest affinity towards NE [38] and exhibit widespread expression in regions associated with decision-making [39, 40].

**Fig. 4:**
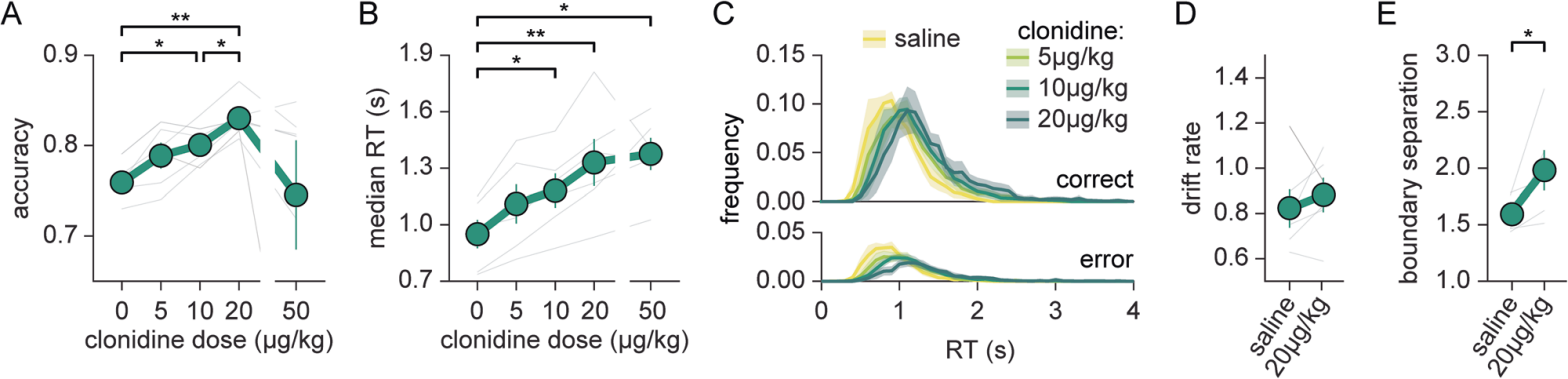
Activation of α2 adrenergic receptors increases boundary separation. **A** Mean accuracy ( ± SEM); *n* = 6. The thin line represents one animal. Paired *t*-test with Bonferroni correction: saline vs. 10 μg/kg clonidine, *t*_(5)_ = 6.386, *p* = 0.014; saline vs. 20 μg/kg clonidine, *t*_(5)_ = 8.304, *p* = 0.004; 10 μg/kg clonidine vs. 20 μg/kg clonidine, *t*_(5)_ = 6.095, *p* = 0.017; *p < 0.05, **p < 0.01. **B** Median reaction time ( ± SEM). Thin line represents one animal. Paired *t*-test with Bonferroni correction: saline vs. 10 μg/kg clonidine, *t*_(5)_ = 7.119, *p* = 0.008; saline vs. 20 μg/kg clonidine, *t*_(5)_ = 5.682, *p* = 0.024; saline vs. 50 μg/kg clonidine, *t*_(5)_ = 5.616, *p* = 0.025; *p < 0.05, **p < 0.01. **C** RT distributions (0.1 s bins) ( ± SEM) in correct (top) and error trials (bottom) following administration of saline or clonidine (various doses). **D** Mean fitted drift rate ( ± SEM) and **E** mean fitted boundary separation ( ± SEM) from DDM under saline and 20 μg/kg clonidine conditions. Paired *t*-test, *t*_*(*5)_ = 2.653, *p* = 0.045; *p < 0.05.

To determine the role of α2-ARs in the shift in speed-accuracy tradeoff, we systemically administered the α2-AR agonist, clonidine (5, 10, 20, 50 μg/kg; see *Methods*). In comparison to saline injections, clonidine administration showed a dose-dependent increase in accuracy, with a peak effect at intermediate doses (20 μg/kg; Fig. 4A). Similar to LC-NE activation, increases in accuracy were coupled with a slowing of RT (Figs. 4B, C and S7) and DDM fits revealed a selective increase in boundary separation (Fig. 4D).

At the highest dose (50 μg/kg), clonidine impaired performance, as evidenced by longer reaction times without further improvements in accuracy (Fig. 4A, B). This dose also led to a reduction in overall activity, as measured by fewer trials completed per session and prolonged initiation time, which may indicate sedative effects (Fig. S7F, G). Together, these data indicate that moderate activation of α2-ARs mimics the effects of LC-NE activation on shifting the speed-accuracy tradeoff and suggest that the LC may alter decision thresholds via the release of NE and activation of α2-ARs.

### Manipulations of LC-NE change task engagement

Past research points towards an involvement of LC-NE in task engagement [9, 41, 42], suggesting its role may extend beyond shifting the speed-accuracy tradeoff and elevating thresholds during decision-making. In this task, engagement can be assessed by initiation time: the latency to initiate a new trial (Fig. 1A).

**Fig. 5:**
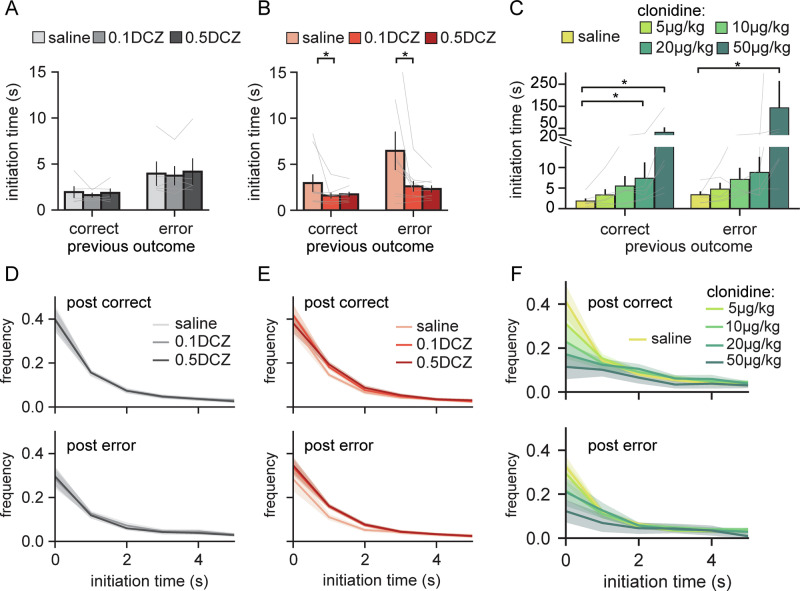
Manipulations of LC-NE change task engagement. Median initiation time (Mean ± SEM) by previous trial outcome (correct vs. error). Each line represents one animal. **A** control group, *n* = 5. **B** excitatory group, *n* = 9. Wilcoxon signed-rank test: saline_correct_ vs. 0.1DCZ_correct_, *p* = 0.023; saline_error_ vs. 0.1DCZ_error_, *p* = 0.035. **C** clonidine group, *n* = 6. Wilcoxon signed-rank test: saline_correct_ vs. 20 μg/kg clonidine_correct_, *p* = 0.031. saline_correct_ vs. 50 μg/kg clonidine_correct_, *p* = 0.031. saline_error_ vs. 50 μg/kg clonidine_error_, *p* = 0.031. *p < 0.05 (uncorrected). Initiation time distributions (bin size = 1 s; mean ± SEM) following correct (top) or error (bottom) trials in **D** control, **E** excitatory, and **F** clonidine groups. The distribution aligns to the left edge of each bin, such that 0 represents the frequency from 0-1s.

Compared to saline injections, DCZ-driven LC-NE activation significantly shortened initiation times; an effect that was not observed in the control (mCherry) group (Fig. 5A, B). As trial initiation is typically faster following rewarded trials [43], reduced initiation times following LC-NE activation could simply reflect the higher accuracy observed in this condition (Fig. 3). Instead, initiation times were shorter upon LC-NE activation following both correct and error trials, indicating a simultaneous effect of LC-NE stimulation in shifting the speed-accuracy tradeoff and enhancing task engagement (Fig. 5A).

Thus, activation of LC-NE neurons induced a non-trivial pattern of results: slower RTs during the stimulus period but faster initiation latency at trial onset. This suggests that slower RTs after LC-NE activation are not the mere consequence of nonspecific motor slowing. Instead, these observations point to a role for the LC-NE system in flexible adjustments of the deliberative process, including the elevation of decision thresholds.

We observed a different pattern of results following clonidine administration. By comparison to saline injections, administration of clonidine slowed initiation times (Fig. 5C, F); an effect that scaled with dose (Fig.  S7). Thus, activation of α2-ARs had the opposite effect on trial initiation compared with direct LC-NE activation. Taken together, these observations suggest that enhancement of task engagement by LC-NE may depend on circuit mechanisms involving receptor classes beyond α2-ARs.

## Discussion

In this study, we investigated the role of LC-NE neurons in setting the balance between speed and accuracy during decision-making under uncertainty. We found that chemogenetic activation of LC-NE neurons drives an increase in decision thresholds and that this change could be reproduced by systemic administration of clonidine. These results suggest a new role for the LC-NE system in decision-making and implicate α2-ARs in the regulation of speed and accuracy during choice. However, to fully account for the behavioral effects of α2-ARs, it is important to thoroughly consider how systemic clonidine differs mechanistically from the chemogenetic perturbation (Fig. S8).

Activation of hM3Dq increases LC–NE firing rates [32] and NE release [44], thereby engaging a broad set of adrenergic receptors, including α1, α2, and β-ARs [45], and possibly other receptors. Like NE, clonidine can directly bind and activate α2-ARs [46], but its effect on other adrenergic receptors is more complex [47] (Fig. S8A). α2-ARs are expressed on LC-NE neurons themselves, where they function as autoreceptors [48]. Activation of these “presynaptic” α2-ARs suppresses LC firing and reduces NE release [49]. As a result, systemic clonidine administration not only activates postsynaptic α2-ARs but is also expected to reduce overall NE levels, thereby decreasing activation of α1- and β- ARs and potentially dopamine receptors [37] throughout the nervous system (Fig. S8B).

Within this framework, the opposing behavioral effects of LC-NE activation and clonidine can be reconciled by considering clonidine’s inhibitory actions at presynaptic α2 autoreceptors within the LC. For example, reduced activation of α1-and β- ARs may be responsible for a reduction of task engagement, as assessed by increased initiation time and a reduction in the number of trials completed. Thus, clonidine’s behavioral effects may reflect a balance between postsynaptic α2-mediated increases in decision threshold and LC autoreceptor–mediated reduction of NE. We note that this autoinhibitory mechanism is dose-dependent and can progressively reduce overall NE level [50] in both the brain and the sympathetic nervous system and produce sedative effects at high doses [51]. Thus, the overall reduction in task engagement observed at the highest levels of clonidine used in this study (50 μg/kg) may be explained by a decrease in arousal caused by inhibition of LC-NE neurons.

Another important caveat to consider is the off-target effect of clonidine. In addition to its action at the α2-AR, clonidine also acts as an agonist at imidazoline receptors [52–54]. Although this interaction occurs with lower affinity (K_D_ = 51 nM [55]) compared with α2-ARs (K_D_ = 1–5 nM [56]), activation of the imidazoline receptor could partially account for the different effects of clonidine compared with LC-NE stimulation.

These differences aside, the common mechanistic endpoint for LC-NE stimulation and clonidine administration is the activation of α2-ARs, and viewed through this lens, our results suggest that α2-ARs may positively regulate decision threshold.

Future experiments will be required to more thoroughly characterize the relationship between NE, α2-ARs, and decision threshold. One direction is a systematic pharmacological dissection using receptor-selective compounds to test the necessity and sufficiency of distinct adrenergic receptors. For example, administration of selective α2-AR antagonists (e.g., atipamezole, yohimbine) could be used to assess whether blocking of α2-ARs is sufficient to reduce decision thresholds. Beyond α2-ARs, the role of α1- and β- receptors could be investigated. β-ARs are particularly intriguing as prior human studies indicated that the β antagonist propranolol can alter the amount of evidence gathered prior to choice [18], suggesting that β-ARs may also contribute to decision threshold regulation.

One advantage of a pharmacological approach is its translational potential, as similar manipulations can be implemented in both rodents and humans. To facilitate this type of translational research, we recently developed a gamified human version of the perceptual task used in this study [23]. Critically, the sensory statistics, task structure, and non-verbal training pipeline are closely matched across the human and rodent versions, enabling direct quantitative comparisons of behavior and model-derived parameters, including decision thresholds. This cross-species framework provides an avenue for testing conserved neuromodulatory mechanisms of decision making and for bridging mechanistic insights from animal models to human cognition.

An alternative experimental direction that leverages the particular strengths of the rodent model is to probe the specific cell types and circuits that mediate the LC’s influence on decision thresholds. Prevailing theory emphasizes LC heterogeneity [12, 57–59], including cell-type specific [60, 61] or projection-specific populations [62, 63], which may differentially influence cognition.

Recent findings suggest how specific projections of LC-NE neurons may regulate decision thresholds. In particular, the superior colliculus (SC), a region implicated in the control of decision thresholds [7], is innervated by the LC-NE neurons [64, 65] and abundantly expresses α2-ARs [39, 66]. Direct suppression of SC neurons, by NE binding to α2-ARs, could delay burst activity that reflects decision commitment. Alternatively, α2-ARs could influence decision thresholds by acting in regions upstream of the SC, such as the cortex [65] or the substantia nigra (SN) [67]. When cortical drive is weak, SN inhibition dominates, suppressing SC activity and delaying commitment. Because adrenergic receptors are enriched in long-range pyramidal neurons of the frontal cortex [68], activation of α2-ARs could reduce excitatory outputs to the SC, thereby increasing decision thresholds. In both scenarios, LC-NE signaling may gate the timing of decision commitment via its influence on SC excitability.

Future experiments in animal models could extend this framework to identify region-specific mechanisms underlying decision threshold regulation. For example, combining local pharmacological manipulations with projection-specific activation of LC–NE neurons could establish whether α2-adrenergic signaling within candidate target regions, such as the SC, is necessary for decision threshold modulation.

Previous models of LC-NE function have emphasized its role in a range of cognitive processes, including attention [15, 69], working memory [70–72], and behavioral flexibility [11, 41, 73–75]. During perceptual decision-making, LC-NE has also been linked to modulation of sensory gain [15, 76–78]; an effect that is typically captured by changes in the drift rate within the DDM [69]. In this study, we observed no significant change in drift rate but instead a change in decision thresholds. Our free-response task emphasized speed-accuracy tradeoff rather than fine sensory discrimination [16, 69], and the use of highly salient stimuli in our task may have reduced our ability to detect LC-NE-dependent changes in sensory gain. Another possibility is that LC-NE may differentially regulate distinct aspects of perception and decision-making depending on the spatio-temporal pattern of neuromodulator release [9, 79]. For instance, decision thresholds may be influenced by tonic LC activity, whereas phasic activity may preferentially support the regulation of sensory processes [15, 77, 80].

## Conclusions

By combining chemogenetic, pharmacological manipulation and behavioral modeling, we discovered that direct activation of the LC-NE system increases decision thresholds; an effect likely mediated through NE release and binding of α2-ARs. We also found that direct LC-NE activation promotes task engagement, whereas α2-ARs produced the opposite effect. Together, this work provides a clear behavioral link between LC-NE signaling and decision threshold, offering a new insight into how LC-NE shapes cognition and complex behavior.

## Supplementary information

Supplementary Figures

## Data Availability

All data will be provided upon request.
