# Supplementary material for: Regulation of the decision threshold by the locus coeruleus
Source: Neuropsychopharmacology. 2026 Apr 13;51(9):1680–9. doi: 10.1038/s41386-026-02399-x (PMC13389323; doi:10.1038/s41386-026-02399-x)
Supplement: Supplementary file 1 — Supplementary Figures [file 41386_2026_2399_MOESM1_ESM.pdf]

Supplementary figures

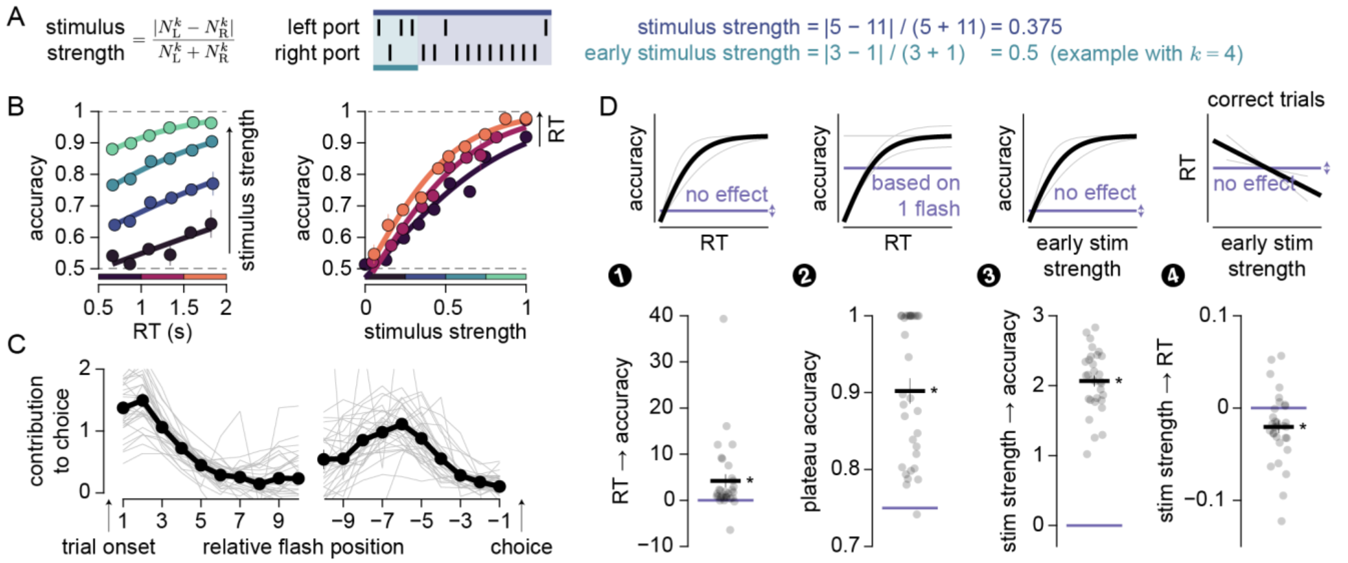

Supplementary Figure 1. Behavioral signatures of sensory evidence accumulation in rats performing the task.

(A) The stochastic nature of stimulus presentation yields trials with different stimulus strengths, quantified by the proportion of flashes pointing towards the same location (irrespective of left or right). Stimulus strength can be computed on all the flashes (dark blue) or restricted to the first *k* flashes (light blue; here with $k = 4$) to reflect early commitment to choice.

(B) Mean ± SEM choice accuracy reflects the combined influence of stimulus strength (blue color coded) and reaction time (RT; red color coded). Thick lines correspond to logistic regression fits and bin limits are shown as colored squares at the bottom of each plot

(C) The relative contribution (A.U.) of individual flashes (left or right) delivered at different position relative to trial onset (left) or choice (right) is estimated using a logistic regression. Thin individual lines correspond to individual rats.

(D) Coefficients from logistic (1-3) and linear (4) regression models (depicted in top row) indicate that accuracy increases with RT (1; vs. 0: $t_{(30)} = 2.97$, $p = 0.006$; same as Fig. 1D), plateau accuracy is larger than expected from a single flash strategy (2; vs. 75%: $t_{(30)} = 9.25$, $p < 0.001$), accuracy increases with early stimulus strength (3; $k = 4$; vs. 0: $t_{(30)} = 26.4$, $p < 0.001$), and RT accelerates with early stimulus strength (4; $k = 4$; vs. 0: $t_{(30)} = −3.00$, $p = 0.005$). * $p < 0.05$.

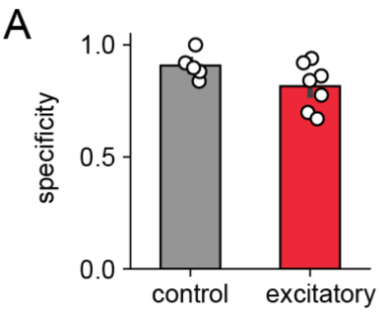

Supplementary Figure 2. Specificity of PRSx8 constructs

(A) Quantification of the specificity of AAV9-PRSx8-mCherry (control) and CAV-PRSx8-hM3Dq-mCherry (excitatory). Specificity is calculated by proportion of the mCherry+ neurons that are also TH+.

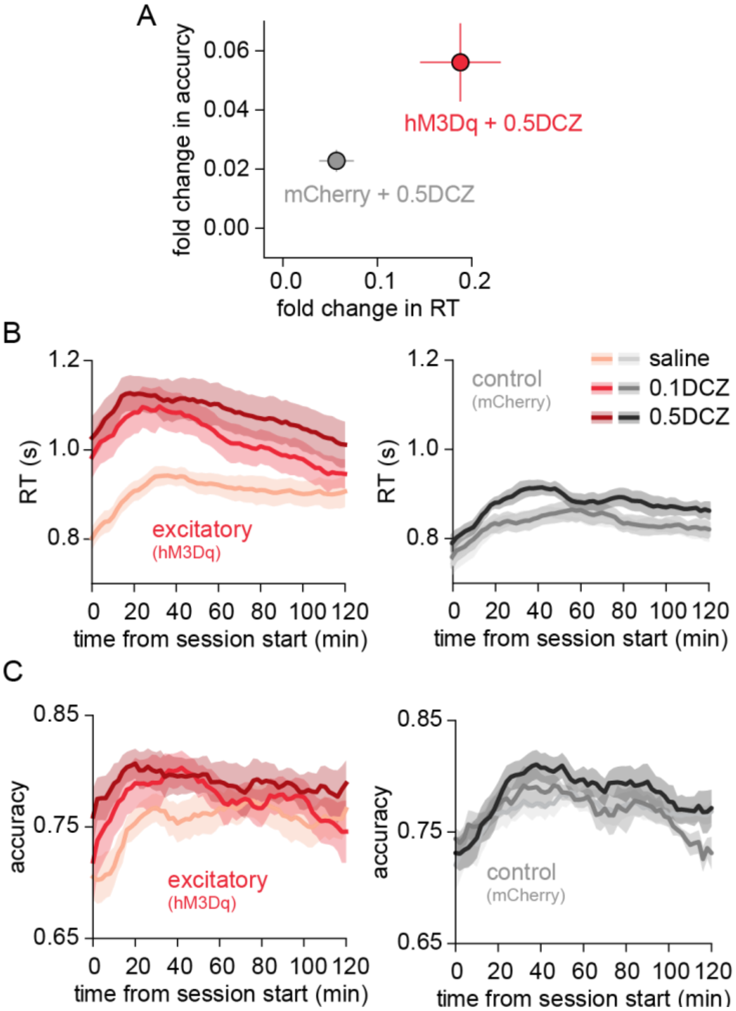

Supplementary Figure 3. Chemogenetic excitation induced a greater change in behavior than DCZ alone.

(**A)** Proportional change of RT and accuracy from saline condition in 0.5mg/kg DCZ in excitatory (red) and control groups (gray).

(B) Rolling average of RT using a 20-minute window across session in excitatory group (left) and control (right). Shaded area represents SEM across animals.

(C) Rolling average of accuracy using a 20-minute window across session in excitatory group (left) and control (right). Shaded area represents SEM across animals.

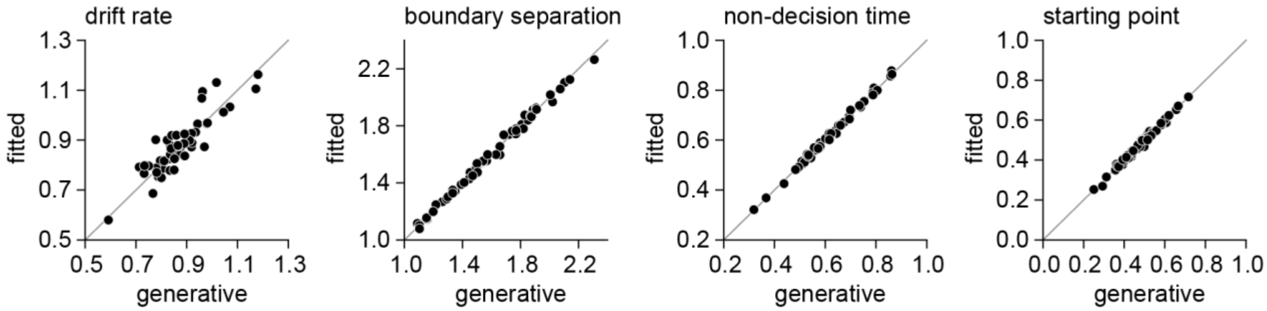

Supplementary Figure 4. DDM parameter recovery and goodness of fit estimates.
Model recovery of generative parameters across 50 artificial agents, each with 2000 trials.

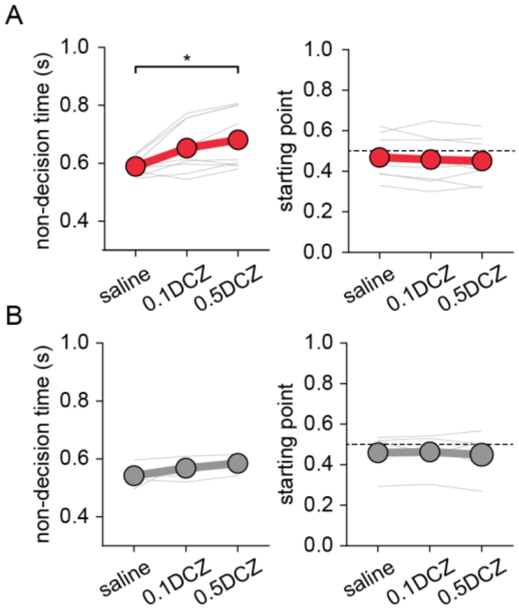

Supplementary Figure 5. DDM estimates of non-decision time and starting point.
(A) Excitatory group. Paired *t*-test with Bonferroni correction, saline vs. 0.5DCZ: $t_{(8)} = 3.265$, $p = 0.034$. * $p < 0.05$
(B) Control group.

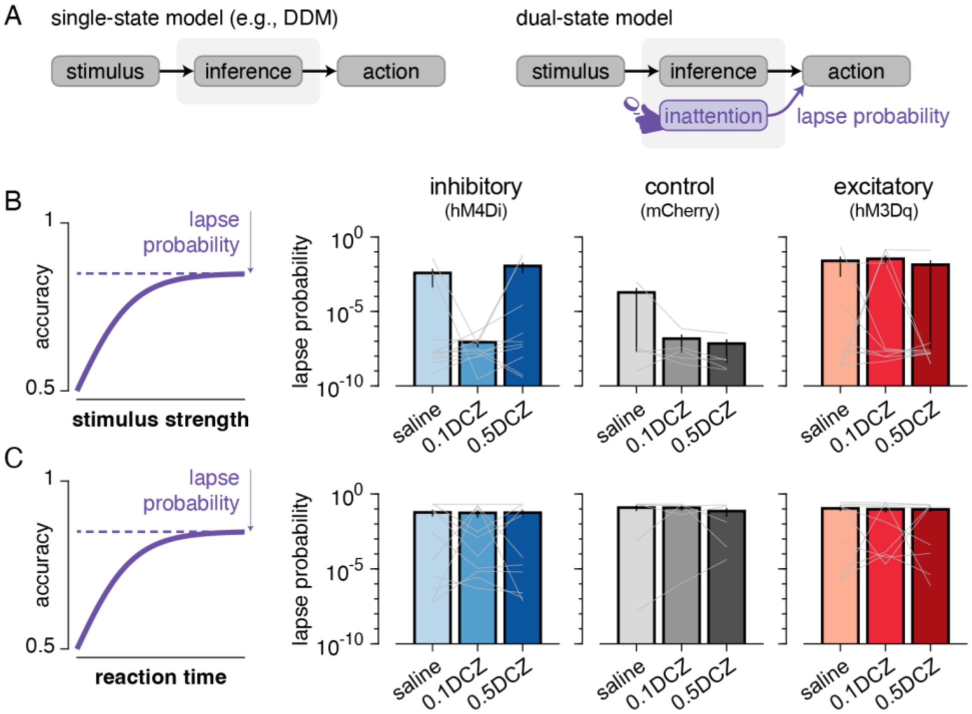

Supplementary Figure 6. **LC-NE manipulations did not detectibly shift attentional allocation.**
(A) Schematics depicting a single-state model in which all choices are stimulus-informed (left) vs. a dual-state model in which choices are either stimulus-informed or made randomly (right). In the dual-state model, the frequency with which random choices are made is controlled by lapse probability parameter.
(B) Lapse probability is estimated based on a version of the dual-state model relating accuracy to stimulus strength (One-way ANOVA, all $p > 0.18$).
(C) Same as panel B but from a version of the dual-state model relating accuracy to reaction times (One-way ANOVA, all $p > 0.59$).

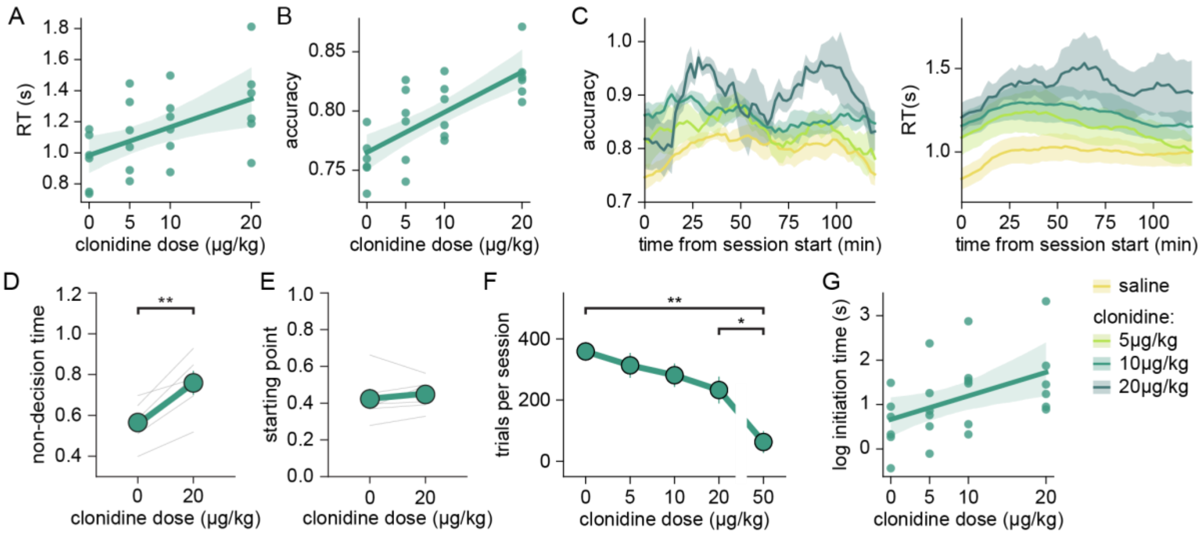

Supplementary Figure 7. **Effect of clonidine, an α2 AR agonist, on the task.**
(A-B) Linear regression model was fitted to **(A)** RT, R^2 = 0.28, p = 0.008 and **(B)** accuracy, R^2 = 0.48, p < 0.0001, across different clonidine dosages. Each dot represents median RT or median accuracy of a single rat across sessions in the experiment. n = 6.
(C) Rolling average of accuracy (left) and RT (right) using a 20-minute window across sessions. Shaded area represents SEM across animals.
(D) Estimated non-decision time for saline and 20μg/kg clonidine from DDM. Each line represents individual animal. Paired t-test, saline vs. 20μg/kg clonidine: $t_{(5)}$ = 5.934, p = 0.002, ** p < 0.01.
(E) Estimated starting point for saline and 20μg/kg clonidine from DDM.
(F) Average number of trials per session across animals. Paired t-test with Bonferroni correction, saline vs. 50μg/kg clonidine: $t_{(5)}$ = 8.775, p = 0.003. 20μg/kg vs. 50μg/kg clonidine: $t_{(5)}$ = 5.251, p = 0.033 * p < 0.05, ** p < 0.05. **(G)** Linear regression fit of log initiation time across rats. Each dot represents individual rats. R^2 = 0.206, p = 0.026.

A

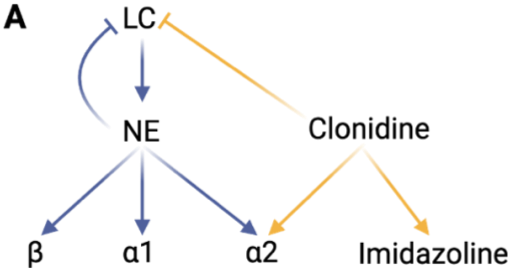

B

	DCZ + HM3Dq	Clonidine
β	+	-
α1	+	-
α2	+	+

Supplementary Figure 8. Conceptual model: Expected molecular and circuit level effects of clonidine and LC-NE stimulation.

(A) Schematic of the network of interrelated effects of NE and clonidine on downstream receptors. Arrows indicate stimulation, T-junctions indicate suppression. Blue lines indicate effects of LC-NE system, yellow lines indicate effects of systemic clonidine. **(B)** Table of expected receptor activation patterns following either LC-NE stimulation with DCZ+ HM3Dq (left) or systemic clonidine (right). + indicates increase and - indicates a decrease in receptor activation relative to baseline. Note that in this model, β and $\alpha 1$ activation is reduced by suppression of NE release via activation of $\alpha 2$ autoreceptors on LC-NE neurons.
